# Pre-injection of saline on the anterior scalene muscle reduced brachial plexus nerve block-induced hemidiaphragmatic paralysis

**DOI:** 10.3389/fmed.2025.1732111

**Published:** 2026-01-22

**Authors:** Yuang Fang, Hong Yu, Beilin Hu, Guo Mu, Huangjun Tang, Yingying Zhang, Jun Zhou

**Affiliations:** 1 Department of Anesthesiology, The Affiliated Hospital of Southwest Medical University, Luzhou, China; 2 Anesthesiology and Critical Care Medicine Key Laboratory of Luzhou, Southwest Medical University, Luzhou, China; 3 School of Basic Medical Sciences, Southwest Medical University, Luzhou, China; 4 Department of Academic Affairs, Clinical Medical College, Southwest Medical University, Luzhou, China

**Keywords:** brachial plexus block, cadaver, hemidiaphragmatic paralysis, local anesthetics, phrenic nerve

## Abstract

**Objective:**

This study aimed to evaluate the effect of pre-injecting normal saline over the anterior scalene muscle (ASM) on the incidence of hemidiaphragmatic paralysis (HDP) following an interscalene brachial plexus block (ISB).

**Methods:**

In this randomized controlled trial, 60 patients selected to upper extremity surgery were divided into two groups: the ASM group (10 ml saline pre-injection followed by 10 ml 0.25% ropivacaine ISB) and the control group (ISB alone). Diaphragmatic excursion (DE) was measured via ultrasonography at baseline and 10, 20, and 30 min post-block during rest and forced breathing. HDP was defined as follows: partial HDP was defined as a ≥ 25% reduction, and complete HDP as a ≥ 75% reduction, in DE from the baseline measurement. Additional Cadaveric dissections assessed anesthetic spread.

**Results:**

At 30 min, the ASM group had significantly lower HDP incidence than controls (rest breathing: 16.6% vs. 76.7%, *p* < 0.001; forced breathing: 10% vs. 70%, *p* < 0.001). DE reduction was also milder in the ASM group (*p* < 0.001). No statistically significant differences were found between the ASM and control groups at post-operation (95% CI:−11.8 to 22.3; *P* = 0.551). Fewer ASM patients experienced hypoxemia (3.3% vs. 26.6%, *p* = 0.027) or hoarseness (0% vs. 20.2%, *p* = 0.024), with equivalent analgesia (*p* > 0.05). Cadaveric studies confirmed that saline blocked anesthetic spread toward the phrenic nerve.

**Conclusion:**

ASM saline pre-injection significantly reduces HDP after ISB without compromising analgesia. This technique may broaden ISB use in patients with respiratory limitations by minimizing diaphragmatic dysfunction.

## 1 Introduction

With the widespread adoption of the enhanced recovery after surgery (ERAS) concept, regional nerve block techniques have emerged as a cornerstone of multimodal perioperative analgesia (1). The interscalene brachial plexus block (ISB), recognized as the gold standard for shoulder arthroscopy analgesia, demonstrates significantly superior analgesic efficacy compared to intravenous analgesia and oral opioids (2), while also facilitating early postoperative motor function recovery (3). However, due to the close anatomical proximity between the brachial plexus and phrenic nerve, ISB frequently results in ipsilateral phrenic nerve blockade, consequently inducing diaphragmatic dysfunction with a significantly elevated incidence, This effect is particularly pronounced when the local anesthetic volume exceeds 15–20 ml, with up to 100% of patients developing hemidiaphragmatic paralysis (HDP) (4).

HDP can induce transient or prolonged diaphragmatic paralysis and weakness during the perioperative period (5). Since the diaphragm is the most important respiratory muscle and accounts for approximately 60% of respiratory function, HDP following nerve block in patients with preoperative respiratory insufficiency often leads to decreased oxygen saturation, hypoxemia, or even dyspnea and postoperative respiratory failure due to impaired diaphragmatic compensation. This adversely affects patient recovery and contradicts the principles of ERAS (6, 7).

Ultrasound-guided nerve block techniques have significantly improved puncture accuracy and optimized local anesthetic distribution through real-time visualization of target nerves and surrounding anatomical structures, thereby markedly reducing the incidence of HDP (8, 9). However, studies have demonstrated that even when using ultrasound-guided ISB with local anesthetic doses as low as 5 ml, HDP still occurs in 30%–40% of patients (10).

Emerging evidence suggests that saline irrigation may mitigate phrenic nerve palsy complications. Ian O. Fleming’s case report demonstrated that high-volume saline irrigation reduces intraneural local anesthetic concentrations below threshold levels, enabling rapid restoration of phrenic nerve conduction and respiratory mechanics in HDP (11, 12). A study additionally reported that saline injection prevented progression from partial to complete diaphragmatic paralysis when administered within one hour of onset, without compromising analgesia (13), While therapeutic saline administration is effective in reversing established phrenic nerve palsy, there is emerging evidence that prophylactic saline application might provide a protective effect. An early randomized controlled trial demonstrated that perineural saline injection (hydrodissection) during interscalene block reduced the incidence of phrenic nerve palsy (14). A subsequent, more recent RCT corroborated these findings, showing that phrenic nerve hydrodissection significantly lowered the incidence of hemidiaphragmatic paralysis (15). However, the current body of evidence is limited, and techniques vary considerably (e.g., in injection site and volume) across studies with heterogeneous designs. In particular, the specific efficacy of a preemptive saline injection superficial to the anterior scalene muscle, which aims to create a physical barrier prior to local anesthetic deposition, has not been rigorously evaluated in a dedicated randomized controlled trial. Therefore, this study was designed to systematically evaluate the impact of this targeted saline preinjection technique on the incidence of ipsilateral phrenic nerve blockade. Our aim was to establish a clinically practical and effective prophylactic strategy.

This study evaluated the efficacy of ASM saline pre-injection in reducing ISB-induced HDP. The primary endpoint was the incidence of HDP using DE evaluated by ultrasound. Secondary endpoints were the pain severity, perioperative hypoxemia, and procedure-related adverse events. Cadaveric studies were conducted to further characterize the anatomic mechanisms underlying saline-mediated phrenic nerve protection.

## 2 Materials and methods

This study was approved by the Ethics Committee of the Affiliated Hospital of Southwest Medical University (Approval Number: KY2024315) and registered in Chinese Clinical Trial Registry (Approval Number: ChiCTR2500101433). Written informed consents were obtained from all participants.

### 2.1 General information

Patients aged 18 to 65 years scheduled for upper limb surgery under general anesthesia at the Affiliated Hospital of Southwest Medical University between 1 July 2024 and 1 July 2025 were enrolled in this study. All participants received ISB for perioperative analgesia. The study employed a single-blind, randomized controlled design. Following written informed consent for anesthesia, participants were randomly allocated to either the experimental or control group in a 1:1 ratio using a computer-generated randomization sequence.

Inclusion criteria: ASA physical status classification ≤ II; Aged 18 to 65 years; scheduled for upper limb surgery under general anesthesia.

Exclusion criteria: emergency surgery, ASA classification > Grade III, contraindications for nerve block (severe respiratory dysfunction, severe coagulation dysfunction, spinal deformity, systemic or local infection), major organ dysfunction (including liver failure or Child-Pugh score of B or C; glomerular filtration rate < 30 ml.min^–1^; New York Heart Association functional class > II; neurological or psychiatric disorders that may hinder cooperation with quiet or deep breathing exercises), difficult airway or anticipated intubation challenges, peripheral neuromuscular disorders, surgery duration exceed 4 h, diaphragm was not clear on ultrasound (excluding operator factors).

The clinical random control trial was followed by a cadaver study for verification, in which four cadaveric specimens were utilized. Four specimens were male without cervical deformities, trauma history, or neoplastic lesions, and with intact cervical skin. A total of four cadaveric specimens were randomly allocated to either the ASM injection group or the control group, with two specimens in each. The specimens were processed according to standardized protocols using formaldehyde-glycerol-alcohol solution fixation, in full compliance with the ethical guidelines and anatomical laboratory management regulations of Southwest Medical University. Each specimen maintained proper donor correspondence throughout the study.

### 2.2 Methods

Randomization was performed by independent research staff using a SAS-generated 1:1 allocation sequence concealed in opaque, sequentially numbered envelopes. Participants remained blinded to group allocation (ASM pre-injection group vs. saline control group) throughout the study duration. All participants adhered to standard preoperative fasting guidelines and received no premedication. Upon arrival in the operating room, standard monitoring (including ECG, non-invasive blood pressure, and pulse oximetry) was initiated, and peripheral intravenous access was secured. Participants were positioned supine with their heads rotated 45° toward the contralateral side. The operative shoulder was elevated approximately 30° using standardized foam padding. A high-frequency linear-array ultrasound transducer (Mindray L12-3RCs, 3–12 MHz) was initially positioned superior to the clavicle. The transducer was systematically advanced cephalad to identify the carotid artery pulsation at approximately 3–4 cm superior to the clavicular margin. The transducer was then carefully translated laterally to obtain optimal visualization of the anterior and middle scalene muscles and the intervening interscalene groove. The C5–C7 nerve roots of the brachial plexus were identified, exhibiting the characteristic hypoechoic “cluster of grapes” appearance within the hyperechoic connective tissue. The needle insertion point was planned at the C5–C6 nerve root level, adjacent to the anterior border of the anterior scalene muscle.

Intervention group: Under continuous ultrasound guidance, a 22-gauge echogenic needle was advanced perpendicular to the transducer plane, traversing the skin, subcutaneous tissue, and fascial layers until contacting the epimysium of the anterior scalene muscle. Under direct visualization, 10 ml of preservative-free normal saline (0.9% NaCl) was slowly injected perimysially around the anterior scalene muscle adjacent to the brachial plexus, creating a circumferential hypoechoic fluid halo.

Control group: Participants received sham needle placement without fluid injection, maintaining identical procedural timing and transducer positioning.

Both groups subsequently received standardized ultrasound-guided ISB. Using an in-plane approach at the C5–C6 neural junction, 5 ml of 0.25% isobaric ropivacaine was deposited on both the superior and inferior aspects of the brachial plexus (total 10 ml), with real-time confirmation of circumferential local anesthetic spread.

All ultrasound-guided interscalene brachial plexus blocks were performed by experienced anesthesiologists, each of whom had performed more than 100 such procedures prior to the study. This standardization of operator skill was implemented to minimize procedure-related technical and interpretive variability. After block administration, patients were maintained in a supine position. Diaphragmatic excursion measurements were performed by an anesthesiologist blinded to group allocation. Using a convex-array transducer (Mindray, 3.5–5 MHz), the operator positioned the probe at the right subcostal margin along the midclavicular line, angling the beam cranially at approximately 45° to obtain a clear view of the hepatodiaphragmatic interface. The hyperechoic curvilinear signature of the diaphragmatic pleura–lung interface was identified adjacent to the hepatic surface. The hyperechoic curvilinear structure representing the diaphragmatic pleura-lung interface was identified adjacent to the hepatic surface. The M-mode cursor was precisely aligned perpendicular to the diaphragmatic dome’s tangent to optimize measurement reliability. Baseline diaphragmatic excursion (T_0_) was recorded prior to block administration. Subsequent measurements were obtained during: (1) quiet breathing (passive, resting respiration); and (2) deep breathing (maximal voluntary inspiration) at 10 min (T_1_), 20 min (T_2_), and 30 min (T_3_) post-block. Three consecutive respiratory cycles were recorded for each breathing pattern, with mean displacement amplitude derived from automated edge-tracking software (16).

#### 2.2.1 Anesthetic management

General anesthesia was induced with sufentanil (0.3–0.5 ug.kg^–1^), propofol (1–2 mg.kg^–1^), and cisatracurium (0.2 mg.kg^–1^), followed by videolaryngoscopy-guided intubation with a reinforced endotracheal tube (ID 7.0–7.5 mm). Mechanical ventilation was maintained at tidal volume 8 ml.kg^–1^ PBW, positive end-expiratory pressure 5 cmH_2_O, fraction of inspired oxygen 50%–80%, and Inhalation:Exhalation ratio: 1:2, with respiratory rate adjusted to keep PaCO_2_ ≤ 60 mmHg. Anesthesia maintenance consisted of remifentanil infusion (0.1–0.2 μg.kg^–1^.min^–1^), sevoflurane (0.7–1.3 MAC), and intermittent cisatracurium, along with balanced crystalloid infusion (4–6 ml.kg^–1^.h^–1^). Neuromuscular blockade was reversed with neostigmine based on TOF monitoring before post-anesthesia care unit (PACU) transfer for 30-min observation.

For postoperative analgesia, all patients received a patient-controlled intravenous analgesia (PCIA) pump with the following regimen: hydromorphone hydrochloride: 1–2 ug⋅h^–1^; butorphanol tartrate: 0.1–0.2 mg⋅h^–1^; granisetron: 0.1 mg⋅h^–1^.

#### 2.2.2 Cadaveric model validation

Cadaveric specimens were positioned supine with 45° contralateral head rotation. Ultrasound-guided anatomical dissection was performed using a Mindray M9 ultrasound system (Shenzhen, China) equipped with a high-frequency linear-array transducer (L14-5Ns, 5–14 MHz). Following standardized ultrasound gel application, the transducer was initially positioned in the supraclavicular fossa to identify: the hyperechoic common carotid artery; the compressible internal jugular vein.

Systematic superolateral transducer movement along these vascular landmarks facilitated identification of the interscalene groove, bounded by the anterior and middle scalene muscles.

In the ASM group, the fascial plane superficial to the ASM was identified under continuous ultrasound guidance. Using an in-plane technique, 10 mL of 0.9% normal saline containing red dye (1:100,000 dilution) was injected at a rate of 0.5 mL.s^–1^, with fluid dispersion patterns dynamically tracked under ultrasound. The transducer orientation was continuously adjusted to confirm circumferential fluid distribution around the ASM. Subsequently, the ultrasound probe was repositioned under guidance to visualize the C5–6 nerve roots. At this location, 10 mL of 0.25% ropivacaine containing 0.1% methylene blue dye was injected to simulate a standard interscalene brachial plexus block. This simulated block was performed in the control group only. Thirty minutes post-injection, a layered dissection of the neck was performed to assess the extent of dye spread.

### 2.3 Outcome measures

#### 2.3.1 Primary outcomes

Incidence of HDP: the primary outcome, the incidence of HDP, was quantitatively assessed through measurement of DE reduction during both quiet and deep breathing at 30 min following nerve block administration, categorized as: (1) Partial HDP: ≥ 25% reduction in DE compared to baseline; (2) Complete HDP: ≥ 75% reduction in DE compared to baseline; (3) Paradoxical or absent diaphragmatic movement on ultrasound (17–19).

#### 2.3.2 Secondary outcome

(1) Pain assessment methodology

Pain intensity was systematically evaluated using the 11-point numerical rating scale (NRS; 0 = no pain, 10 = worst imaginable pain) at predetermined intervals: post-operation, 12 h (± 1 h), 24 h (± 1 h), and 48 h (± 1 h) post-block. The maximum pain score reported during each assessment window was recorded. For early-discharged participants, structured telephone interviews conducted by blinded research staff obtained NRS scores within protocol-defined time windows.

(2) Analgesia efficacy monitoring

Time to first rescue analgesia was defined as the initial activation of PCIA or administration of supplemental intravenous analgesics postoperatively. Analgesic consumption included both PCIA usage (hydromorphone plus butorphanol) and cumulative rescue analgesic doses (flurbiprofen axetil or tramadol). All drug doses were standardized and analyzed according to the Guidelines for Postoperative Pain Management (20).

(3) Perioperative hypoxemia

During PACU monitoring, arterial blood gas (ABG) analysis was performed according to protocol; hypoxemia was diagnosed using standardized criteria: diagnostic threshold: PaO_2_ < 60 mmHg (sea level, resting state, room air); mild (PaO_2_ 60–79 mmHg): typically asymptomatic at rest; moderate (PaO_2_ 40–59 mmHg): dyspnea on exertion ± mild cognitive impairment; severe (PaO_2_ < 40 mmHg): cyanosis, significant neurological compromise, impending organ failure (21, 22).

(4) Postoperative adverse events (AEs):

General safety: Peripheral nerve blocks are considered low-risk, with an overall complication rate of ∼0.05%. Reported AEs Included: (1) Local hematoma: assessed by visual inspection and ultrasonographic measurement, (2) Nerve injury: nerve conduction studies (NCS) and electromyography (EMG) at 48 h post-block for persistent symptoms, (3) Pneumothorax: chest X-ray (gold standard) or lung ultrasound showing pleural line absence with lung sliding loss, (4) Horner’s syndrome: 1. Ptosis (eyelid droop ≥ 2 mm) 2. Iosis (pupil diameter difference ≥ 1 mm) 3. Anhidrosis (confirmed by starch-iodine test), (5) Hoarseness: GRBAS scale (Grade, Roughness, Breathiness, Asthenia, Strain) assessments were performed by blinded ENT specialists at 30 min post-block (23).

### 2.4 Statistical analysis

The sample size was calculated based on previous literature, which reported that the incidence of diaphragmatic paralysis following ISB can reach 100%. Assuming an incidence rate of 80% for HDP in the control group, we aimed for a 50% reduction in the experimental group. Using a one-sided significance level of 0.025, a power (1-β) of 0.9, and a 1:1 allocation ratio between the control and experimental groups, we determined that each group required 27 patients. Accounting for a 10% dropout rate or exclusions due to failure to meet inclusion criteria, the total sample size was calculated as 60 patients (30 per group).

Data were analyzed using SPSS Statistics version 27.0 (IBM Corp). Continuous variables with normal distribution were presented as mean ± standard deviation (SD) and compared using Student’s *t*-test, while non-normally distributed data were analyzed with non-parametric tests. Categorical variables, including demographic characteristics between ASM and Control groups, were compared using Fisher’s exact test for primary outcomes and other categorical data analyses. All figures were generated using GraphPad Prism software (version 10; GraphPad Software, San Diego, CA, USA).

## 3 Results

### 3.1 General information

**FIGURE 1:**
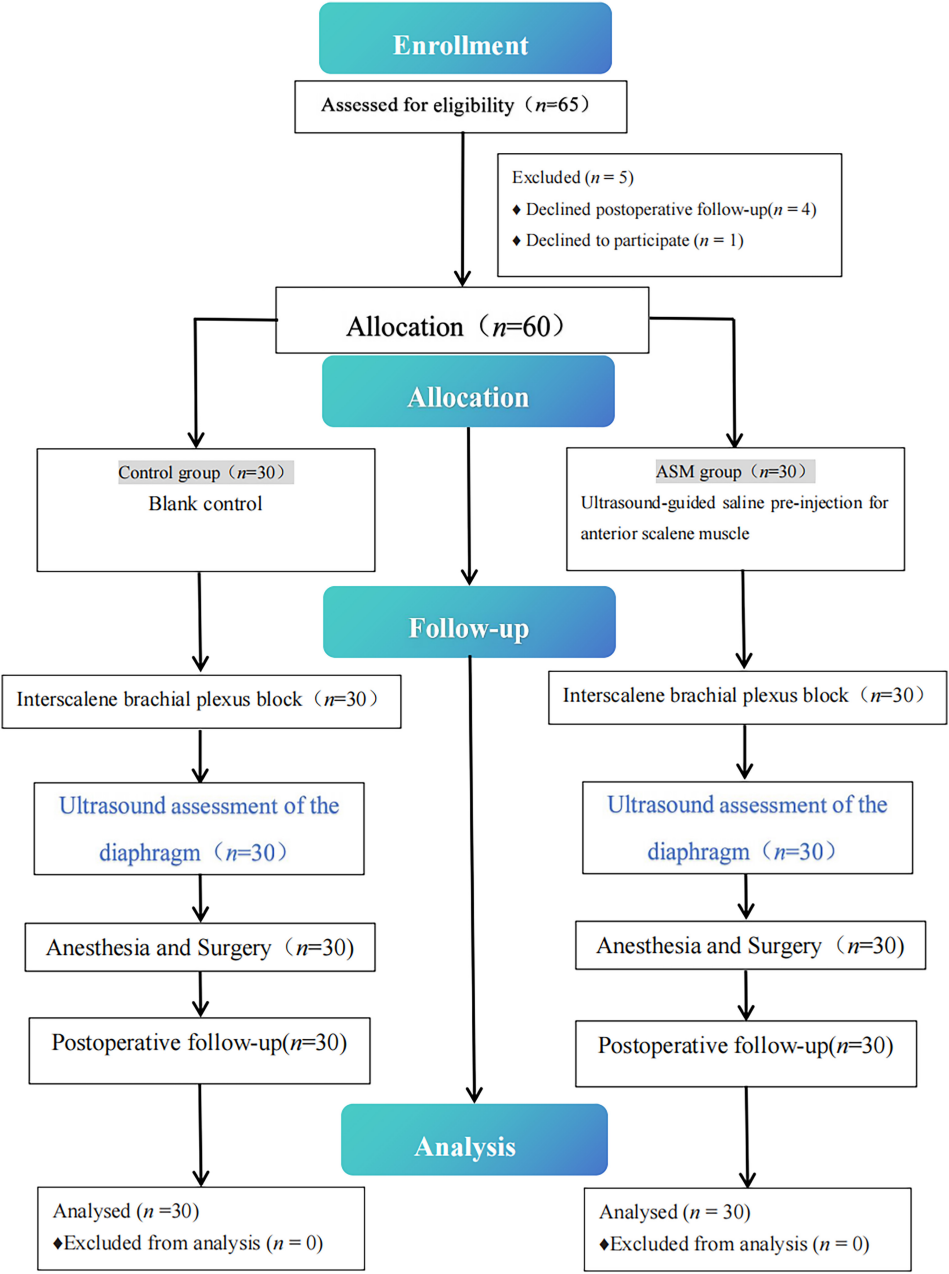
Study now diagram outlining participant eligibility, allocation, and follow-up in the clinical trial.

Figure 1 illustrates the study flowchart. A total of 65 patients were initially enrolled (first enrollment date: 11 July 2024; study completion date: 15 July 2024). After providing informed consent, four patients declined postoperative follow-up and one patient withdrew from further participation. The remaining 60 patients were randomly assigned to two study groups.

As presented in Table 1, no statistically significant differences were found between the two groups in demographic characteristics (sex, age, weight, body mass index [BMI]), American Society of Anesthesiologists (ASA) physical status, surgical site and scope, operative duration, anesthetic drug administration, or postoperative analgesic requirements.

**TABLE 1:** Description of characteristics in patients.

General characteristics	ASM (*n* = 30)	Control (*n* = 30)	*P*-value
**Perioperative factors**			
Gender (male)	17 (57%)	15 (50%)	0.722
Age (years)	53 ± 9.1	50 ± 13.8	0.552
Height (cm)	160.3 ± 6.9	158.5 ± 6.7	0.284
Weight (kg)	60.5 ± 9.1	58.6 ± 10.9	0.452
BMI (kg.m^–2^)	23.5 ± 2.8	23.2 ± 3.3	0.687
Hypertension	12 (40%)	10 (30%)	0.724
Diabetes	2 (6%)	2 (6%)	1
Coronary artery disease	5 (16%)	2 (6%)	0.482
ASA physical status classification			0.652
I (best health)	2 (6%)	3 (10%)	
II	28 (94%)	27 (90%)	
**Surgical data**			
Surgical site (right)	28 (93%)	21 (70%)	0.121
Surgical scope			0.652
Humerus	1 (3%)	4 (13%)	
Shoulder joint	29 (97%)	26 (87%)	
**Duration of surgery (min)**	111.5 ± 26.7	106.7 ± 24.9	0.449
**Consumption of anesthetic agent**			
Remifentanil (ug.kg^–1^)	20.3 ± 7.2	21.3 ± 9.2	0.602
Sufentanil (ug.kg^–1^)	0.37 ± 0.07	0.41 ± 0.11	0.074
Propofol (mg.kg^–1)^	2.5 ± 1.1	2.2 ± 0.9	0.265
Muscle relaxant (mg.kg^–1^)	0.1	0.1	1
Dezocine (mg.kg^–1^)	0.1	0.1	1
**Fluid balance**			
Crystalloid (ml.kg^–1^)	25.2 ± 11.5	23.8 ± 6.5	0.549
Blood loss (ml.kg^–1^)	1.1 ± 11.2	1.2 ± 1.0	0.683
**Consumption of analgesia drugs**			
Hydromorphone (mg.kg^–1^)	0.12 ± 0.029	0.11 ± 0.029	0.647
Flurbiprofen axetil (mg.kg^–1^)	0.64 ± 0.59	0.50 ± 0.59	0.359
Butorphanol tartrate (ug.kg^–1^)	86.5 ± 27.5	88.1 ± 16.2	0.077

Values are number (proportion).

### 3.2 Verification by cadaveric dissection

**FIGURE 2:**
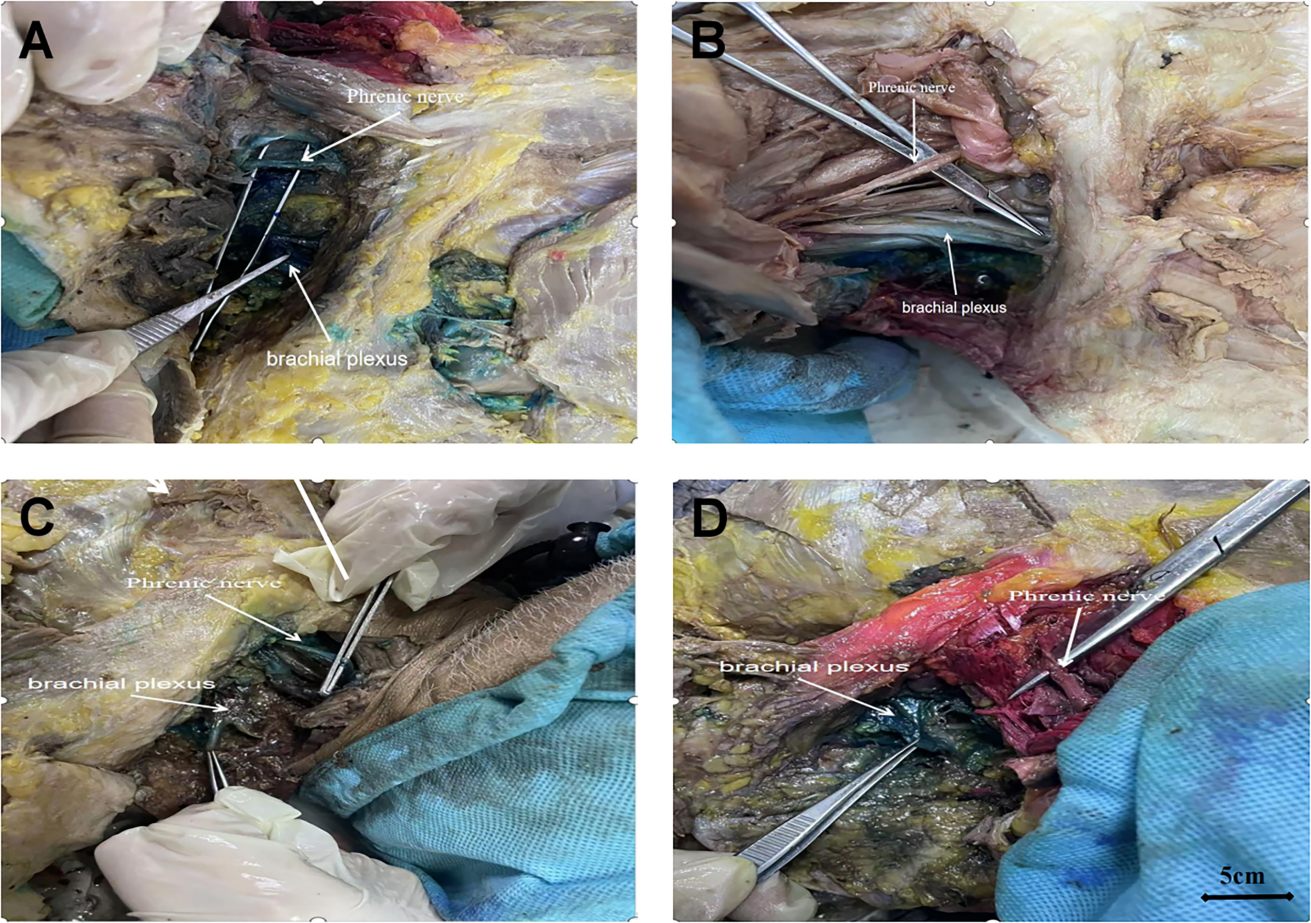
The potential mechanisms underlying saline pre-injection were investigated through a cadaveric study. In panels **(A,C)**, the schematic diagrams illustrate the dispersion pattern of local anesthetic during an interscalene brachial plexus block, demonstrating spread along the ventral surface of the anterior scalene muscle and circumferential encapsulation of the phrenic nerve. In panels **(B,D)**, the pre-injection process in the ASM group was simulated using red dye, with two representative images showing complete circumferential encapsulation of the phrenic nerve along the ventral surface of the anterior scalene muscle. This physical barrier effect effectively prevented subsequent spread of the local anesthetic to the target region during the interscalene brachial plexus block.

Standardized layered dissection (including fat-muscle tissue separation and sternocleidomastoid muscle transection) was performed in four cadaveric specimens. The control group exhibited extensive three-dimensional diffusion (distribution area: 15 cm × 10 cm): longitudinal spread extended from the phrenic nerve pathway along the anterior scalene muscle surface to the supraclavicular brachial plexus, while transverse spread reached from the deep fascia to the lateral vertebral edge, indicating complete permeation of all brachial plexus trunks and branches (Figures 2A, C). The ASM group demonstrated a unique double-staining distribution pattern: methylene blue was confined to the brachial plexus pathway, whereas red dye infiltrated the anterior scalene muscle surface (Figures 2B, D). The results suggest that normal saline forms a physical barrier on the phrenic nerve surface, leading to a significant reduction in methylene blue contact with the phrenic nerve.

### 3.3 Ultrasound-guided phrenic nerve protection

**FIGURE 3:**
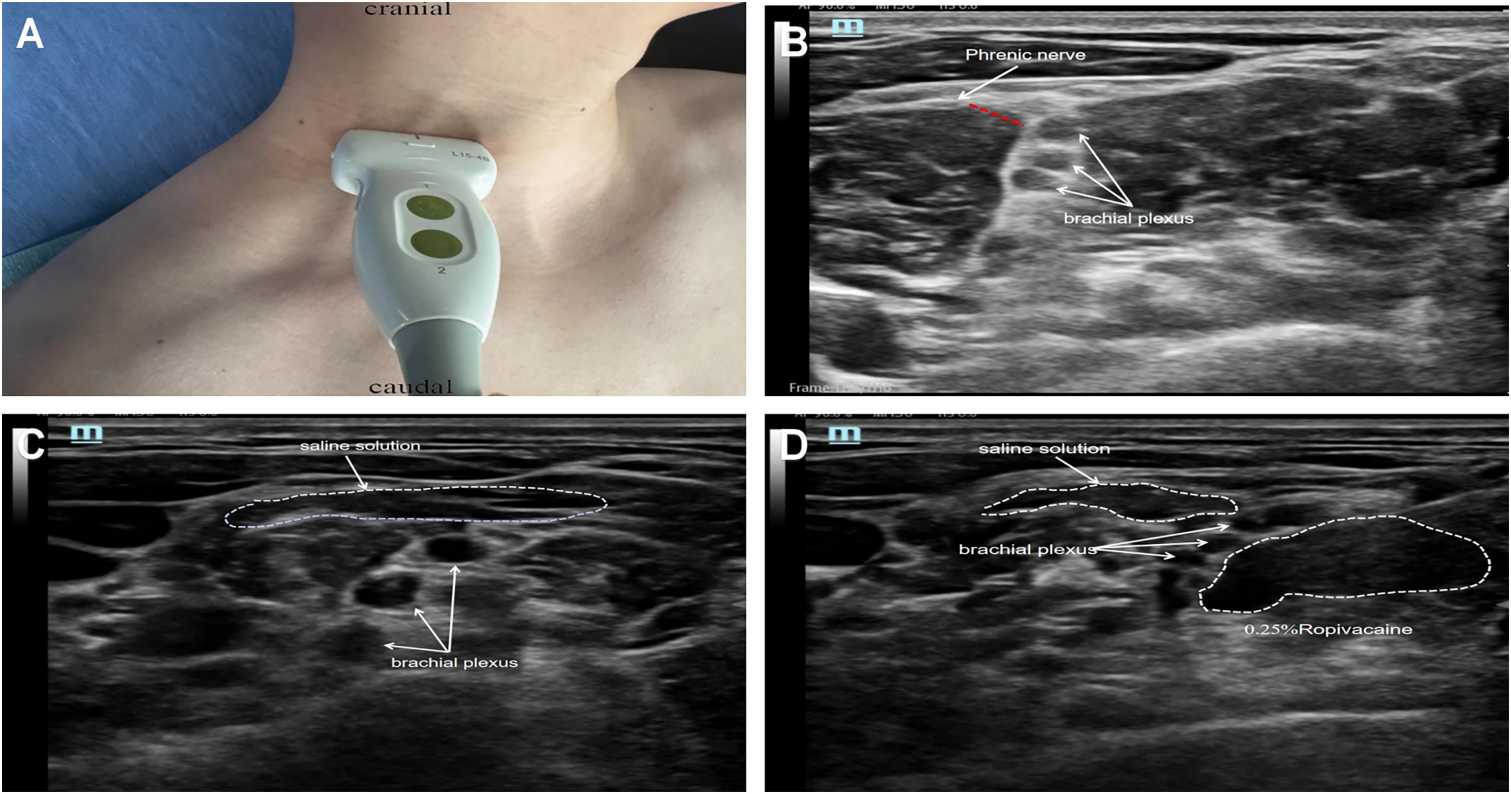
Ultrasound guided saline pre-injection and biracial plexus block. **(A)** Transducer positioning at surface landmark. **(B)** The distribution of the phrenic nerve and brachial plexus can be visualized under ultrasound guidance. **(C)** Normal saline forms a hypoechoic fluid space around the phrenic nerve. **(D)** Normal saline interferes with local anesthetic action through physical blockade and concentration dilution.

In clinical ultrasound, the phrenic nerve and brachial plexus are separated only by a fascial layer (Figures 3A, B). Following pre-injection of normal saline, a hypoechoic fluid-filled space was observed on the anterior scalene muscle surface (Figure 3C). After local anesthetic injection, the anesthetic spread did not extend toward the anterior scalene muscle surface (Figure 3D), which was consistent with our cadaveric findings. Pre-injection of normal saline may exert a protective mechanism via: (1) significant separation between the phrenic nerve and brachial plexus, and (2) creation of a physical barrier or dilution of local anesthetic around the phrenic nerve.

### 3.4 Incidence of hemidiaphragmatic paralysis

**FIGURE 4:**
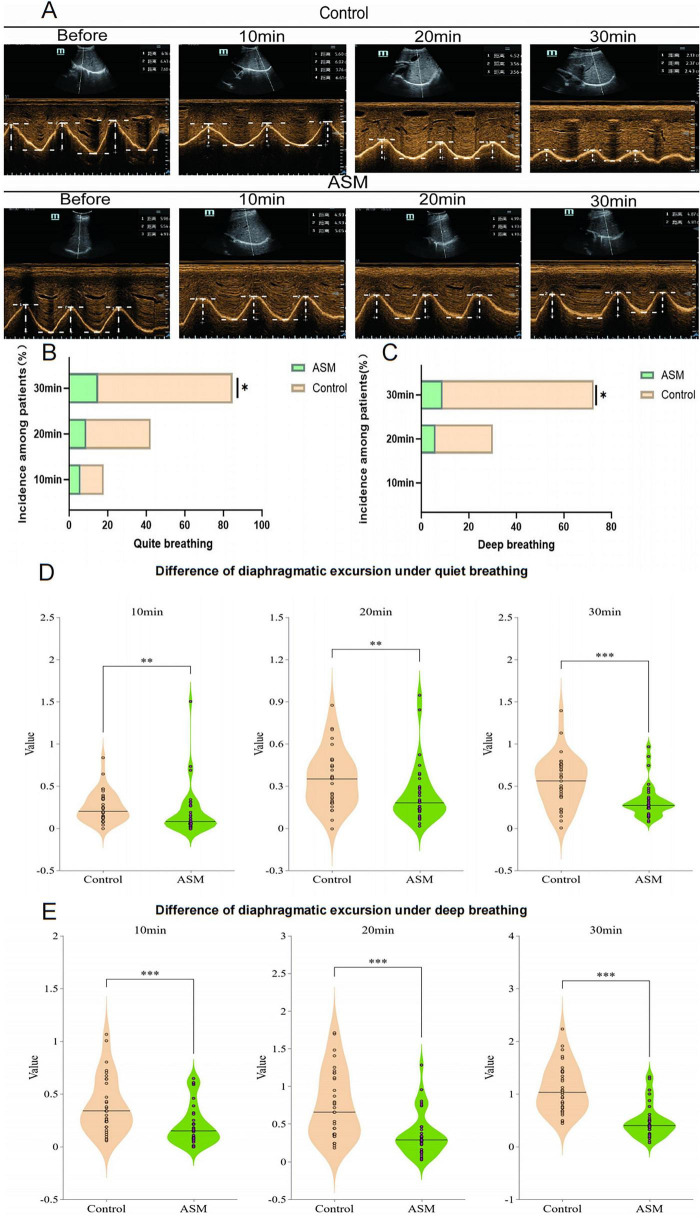
Panel **(A)** provides representative ultrasonographic images acquired during forced respiration, including pre-block baseline and serial post-block assessments at 10-min intervals. Panels **(B,C)** illustrate the comparative incidence rates of hemidiaphragmatic paralysis between the intervention and control groups during quiet and forced respiration, respectively. Panels **(D,E)** depict the temporal changes in diaphragmatic excursion between groups at sequential post-block time points (10, 20, and 30 min). *p < 0.05, **p < 0.01, ***p < 0.001.

At 30 min post-block, under quiet breathing conditions, the ASM group demonstrated a significantly lower incidence of hemidiaphragmatic paralysis (16.6% vs. 76.7%; OR = 0.07, 95% CI 0.04–0.28, *p* < 0.001; Figure 4B). This protective effect was more pronounced during deep breathing (10% vs. 70%; OR = 0.05, 95% CI 0.02–0.22, *p* < 0.001; Figure 4C), demonstrating that normal saline pre-injection significantly reduces the occurrence of hemidiaphragmatic paralysis.

### 3.5 Diaphragmatic excursion difference

Compared to baseline measurements, significant differences in diaphragmatic excursion during quiet breathing were observed at: 10 min post-block (Control: 0.26 ± 0.18 cm; 95% CI: 0.16–0.32 vs. ASM: 0.17 ± 0.22 cm; 95% CI: 0.07–0.254; *P* = 0.02), 20 min post-block (Control: 0.36 ± 0.20 cm; 95% CI: 0.27–0.45 vs. ASM: 0.22 ± 0.19 cm; 95% CI: 0.16–0.29; *P* = 0.008), and 30 min post-block (Control: 0.70 ± 0.42 cm; 95% CI: 0.55–0.85 vs. ASM: 0.35 ± 0.29 cm; 95% CI: 0.25–0.45; *P* < 0.001) (Figure 4D). Similar trends were observed during deep breathing at: 10 min post-block (Control: 0.39 ± 0.25 cm; 95% CI: 0.29–0.48 vs. ASM: 0.18 ± 0.15 cm; 95% CI: 0.12–0.24; *P* = 0.001), 20 min post-block (Control: 0.35 ± 0.21 cm; 95% CI: 0.28–0.42 vs. ASM: 0.22 ± 0.20 cm; 95% CI: 0.14–0.29; *P* < 0.001), and 30 min post-block (Control: 1.06 ± 0.44 cm; 95% CI: 0.88–1.25 vs. ASM: 0.45 ± 0.30 cm; 95% CI: 0.35–0.55; *P* < 0.001) (Figure 4E). Both groups showed progressive reductions in diaphragmatic displacement over time, with the ASM group exhibiting significantly less decline compared to controls (Figure 4A).

### 3.6 Postoperative analgesia

Postoperative analgesia was evaluated using NRS scores at Post-Operation, 12, 24, and 48 h following block administration. No statistically significant differences were found between the ASM and control groups at post-operation (mean difference: 6.0%; 95% CI: −11.8 to 22.3; *P* = 0.551) or 12 h post-block (mean difference: 3.0%; 95% CI: −17.6 to 23.6; *P* = 0.575). Considering ropivacaine’s typical duration of action (8–12 h), the 24- and 48-h pain assessments were not clinically relevant to the nerve block effects (Table 2).

**TABLE 2:** The secondary outcomes.

Secondary outcomes	ASM (*n* = 30)	Control (*n* = 30)	*P*-value
**Pain severity (NRS)**			
Pain scores at post-operation			0.551
Mild (0–3)	24	26	
Moderate (4–6)	6	4	
Severe (7–10)	0	0	
Pain scores at 12 h post-block			0.575
Mild (0–3)	13	15	
Moderate (4–6)	16	15	
Severe (7–10)	1	0	
Pain scores at 24 h post-block			0.547
Mild (0–3)	9	12	
Moderate (4–6)	21	18	
Severe (7–10)	0	0	
Pain scores at 48 h post-block			0.804
Mild (0–3)	13	15	
Moderate (4–6)	17	15	
Severe (7–10)	0	0	
Hypoxemia	1	8	0.032
**Postoperative adverse events**			
local hematoma	2	0	0.492
Nerve injury	0	0	1
Pneumothorax	0	0	1
Hoarseness	0	6	0.024
Horner’s syndrome	0	0	1

DE, diaphragmatic excursion; NRS, numerical rating scale.

### 3.7 Postoperative adverse events

The ASM group exhibited a significantly reduced incidence of PACU hypoxemia compared with controls (24.2% vs. 3.0%; ARR: 21.2%, 95% CI: 0.11–0.47; *P* = 0.027; Table 2). These results indicate that normal saline pre-injection may limit local anesthetic diffusion toward the phrenic nerve, consequently reducing the incidence of hemidiaphragmatic paralysis.

Pre-injection of normal saline significantly decreased the incidence of post-procedural hoarseness (ARR: 18.1%; 95% CI: 0.17–0.44; *P* = 0.024). Within the ASM cohort, two instances of localized swelling were identified. Doppler ultrasound confirmed these as normal saline infiltration sites, with both resolving spontaneously within 24 h without intervention. No statistically significant differences emerged between the groups in other postoperative complications (*P* > 0.05).

## 4 Discussion

The findings of this study demonstrate that a normal saline pre-injection over the ASM significantly reduced the incidence of HDP following ISB. Importantly, the technique preserved postoperative analgesic efficacy, as evidenced by no significant differences in NRS pain scores between the groups. Furthermore, patients in the ASM pre-injection group exhibited statistically significant reductions in the incidence of both postoperative hypoxemia and overall procedure-related adverse events compared to those in the control group.

This study aimed to evaluate the incidence of HDP following ultrasound-guided ISB. The primary study population comprised patients scheduled for shoulder arthroscopy, for which ISB is the standard analgesic technique. To improve the generalizability of the findings, a subset of patients scheduled for humeral surgery was also included. Statistical analysis confirmed that the distribution of surgical types was balanced between the experimental and control groups, and that the type of surgery did not significantly influence the incidence of HDP or other key outcomes, thereby supporting the validity of the study’s conclusions. During participant screening, individuals aged over 65 years were excluded due to age-related respiratory vulnerability. This age group exhibits a higher risk of respiratory compromise, as younger individuals typically compensate for unilateral diaphragmatic paralysis through increased respiratory rate and enhanced excursion of the contralateral hemidiaphragm, thereby maintaining adequate ventilation without dyspnea (24). Elderly patients exhibit reductions in transdiaphragmatic pressure (Pdi) of 20%–41% and an approximately 30% decrease in diaphragmatic contractility. Consequently, the failure of these compensatory mechanisms places elderly patients at a substantially higher risk of perioperative hypoxemia (OR: 3.2; 95% CI: 1.8–5.7) and respiratory failure (25). Consequently, enrollment was restricted to adults aged 18–65 years to mitigate these age-related respiratory risks and minimize the potential for severe perioperative complications. Therefore, the potential efficacy of saline pre-injection in elderly populations warrants future investigation in larger, multicenter trials with adequate statistical power. Traditional clinical indicators for diagnosing diaphragmatic paralysis are associated with significant delays; therefore, we employed diaphragmatic ultrasonography to enable a precise functional assessment. This methodology facilitates the real-time and objective quantification of diaphragmatic kinetics (26). Prolonged non-invasive mechanical ventilation (NIV) induces several detrimental effects on diaphragmatic function, including (1) impaired force-generation capacity, (2) structural muscle fiber damage, and (3) progressive muscle atrophy. Extended durations of mechanical ventilation markedly accelerate diaphragmatic atrophy and contractile dysfunction, a condition termed ventilator-induced diaphragm dysfunction (VIDD). The underlying pathophysiological mechanisms primarily involve (1) activation of the ubiquitin-proteasome system, (2) accumulation of mitochondrial reactive oxygen species (ROS), and (3) upregulation of myostatin (27, 28). A strict 4-h limit for surgical procedures was implemented in this investigation, based on two key physiological considerations: (1) to prevent the activation of mechanical ventilation-induced protein degradation pathways, which typically initiates after 6–8 h; and (2) to minimize cumulative oxidative damage to diaphragmatic sarcolemmal structures. This protocol was designed to maintain the validity of postoperative diaphragmatic function assessment and ensure data integrity.

HDP remains an inherent challenge in achieving surgical analgesia for upper extremity procedures, particularly those involving the shoulder, due to unavoidable anatomical constraints. Specifically, the phrenic nerve courses within 1.8 mm of the brachial plexus at the C6 vertebral level. This proximity accounts for the reported 33% incidence of HDP observed even with minimal local anesthetic volumes (5 ml), as demonstrated in the study by Tedore et al. (29). Although Sun et al. (30) demonstrated that erector spinae plane (ESP) blocks provide effective analgesia while avoiding HDP, the technical complexity of this approach may limit its widespread clinical adoption. While other alternative methods, such as the L-shaped barrier technique described by Liangguang et al. (31), also effectively reduce HDP, our pre-block saline injection demonstrated superior efficacy (16.6% vs. 76.7% incidence; *P* < 0.001). This preservation of diaphragmatic function is particularly valuable for postoperative shoulder rehabilitation, given that early passive and active mobilization following procedures such as rotator cuff repair is essential for preventing joint stiffness. Preserved diaphragmatic function enhances patient tolerance for intensive rehabilitation protocols, thereby reducing the risk of adhesions and optimizing long-term functional outcomes (32).

Cumulative evidence demonstrates a dose-dependent relationship between local anesthetic administration and the incidence of HDP, wherein reduced volumes and concentrations yield significantly lower rates of paralysis (33, 34), Specifically, volumes of ≤ 10 ml of 0.25% ropivacaine maintain effective analgesia while significantly reducing the incidence of HDP, particularly in arthroscopic rotator cuff repair procedures (35). Based on this evidence, a volume of 10 ml of 0.25% ropivacaine was standardized for the ISB procedure in our protocol. Notably, one patient with a massive rotator cuff tear experienced severe pain (NRS ≥ 7), a finding that underscores the necessity for individualized analgesic approaches in complex cases (36).

Pharmacodynamic evidence substantiates our methodological rationale: Grelet et al. (37) established 0.22% ropivacaine as the minimum effective concentration (MEC<sub>90</sub>) for sensory blockade, and the known concentration-dependence of motor blockade duration (38) explains the maintained analgesic efficacy observed with the use of a diluted anesthetic. Building upon this prior evidence and our findings, we propose that the prophylactic saline injection exerts its protective effect through a dual mechanism. First, it creates a physical barrier—visually confirmed by ultrasound and cadaveric dissection—that impedes the cephalad spread of local anesthetic toward the phrenic nerve, which lies on the anterior surface of the ASM. Second, by compartmentalizing the injectate, it confines the diluted local anesthetic (0.25% ropivacaine) around the brachial plexus roots at a concentration exceeding the reported MEC<sub>90</sub> for sensory blockade. This preserves analgesic efficacy while minimizing exposure of the adjacent phrenic nerve to concentrations sufficient to induce motor blockade. This combined action—spatial separation of the phrenic nerve from the anesthetic depot and maintenance of effective sensory blockade at the target site—constitutes a clinically viable strategy to mitigate HDP. This technique complements ERAS multimodal analgesia protocols, which typically incorporate nonsteroidal anti-inflammatory drugs (NSAIDs), acetaminophen, and low-dose opioid regimens. By simultaneously providing effective analgesia and mitigating diaphragm-related complications, this approach enhances patient adherence to early rehabilitation protocols and optimizes the overall efficacy of ERAS programs (39).

Although ISB consistently induces HDP, the clinical implications of this effect require nuanced evaluation. Our data indicate that most patients—particularly younger, healthier individuals—effectively compensate for unilateral diaphragmatic dysfunction without clinically apparent respiratory impairment via well-characterized mechanisms, such as tachypnea and increased excursion of the contralateral hemidiaphragm. However, this presumption of universal tolerance overlooks vulnerable patient populations in whom transient HDP may precipitate serious respiratory complications.

High-risk populations include patients with preexisting respiratory conditions—such as chronic obstructive pulmonary disease (COPD), obstructive sleep apnea (OSA), or obesity hypoventilation syndrome—who exhibit diminished pulmonary reserve. This compromised reserve renders them vulnerable to complications such as HDP-induced hypoxemia or difficulty in weaning from mechanical ventilation (6, 7). Furthermore, older adults experience a progressive, age-related deterioration in diaphragmatic contractility and excursion, which substantially elevates their risk for postoperative pulmonary complications (PPCs).

The functional implications of this are significant. Early postoperative mobilization is a critical component of optimal shoulder rehabilitation, particularly for restoring range of motion and preventing adhesive capsulitis (32). Consequently, HDP-associated respiratory impairment can substantially compromise functional recovery, potentially prolonging rehabilitation timelines by 2–3 weeks in affected patients. Our findings should be interpreted within the context of existing regional anesthesia techniques designed to preserve diaphragmatic function. Several alternative approaches—such as the superior trunk block, low-volume supraclavicular block, and the more recent erector spinae plane block—have been proposed to provide shoulder analgesia while minimizing phrenic nerve involvement. Although these techniques differ in their anatomical targets and procedural complexity, they share the common goal of decoupling brachial plexus analgesia from diaphragmatic impairment. The saline pre-injection technique described here introduces a distinct mechanistic principle: establishing a physical barrier at the critical anatomical interface between the phrenic nerve and the brachial plexus. A key practical advantage is that this strategy can be integrated into conventional interscalene brachial plexus block practice without altering standard sonographic landmarks or needle trajectories, thereby enhancing its clinical adaptability. While supplemental oxygen mitigates symptomatic hypoxemia, it does not address the underlying pathophysiology of local anesthetic-induced phrenic nerve palsy. In contrast, the prophylactic saline pre-injection technique investigated in this study—which specifically targets the anterior scalene muscle surface prior to local anesthetic deposition—demonstrated a significant reduction in HDP incidence.

In addition to reducing the incidence of HDP, our study revealed significant secondary benefits associated with ASM saline pre-injection. The significantly lower incidence of postoperative hypoxemia (26.6% vs. 3.3%; ARR 21.2%, 95% CI 0.15–0.51, *P* = 0.032) and hoarseness (ARR 18.1%, 95% CI 0.17–0.44, *P* = 0.024) in the intervention group underscores the multisystem protective effect of this technique. These findings align with the anatomical rationale that limiting anesthetic spread along the anterior scalene muscle surface shields the phrenic nerve and reduces inadvertent involvement of proximate neural structures, such as the recurrent laryngeal nerve. The reduction in hypoxemia is clinically significant, as it directly mitigates a common and potentially serious complication, particularly in patients with limited respiratory reserve. This benefit supports early mobilization and ERAS protocols. While previous studies have primarily focused on diaphragmatic outcomes, our results indicate that targeted perineural hydrodissection confers broader protective benefits. This reinforces the technique’s value in enhancing perioperative safety and patient comfort without compromising analgesia.

This technique represents a significant clinical optimization, as the saline pre-injection offers proactive prevention of HDP while maintaining analgesic efficacy equivalent to that of the standard block. The ASM group exhibited clinically meaningful reductions in both hypoxemia (26.6% vs. 3.3%; ARR: 21.2%; 95% CI: 0.15–0.51; *P* = 0.032) and hoarseness (ARR: 18.1%; 95% CI: 0.17–0.44; *P* = 0.024), which demonstrates multisystem benefits beyond diaphragmatic preservation. These outcomes align with and enhance ERAS principles by facilitating earlier and more aggressive rehabilitation protocols.

### 4.1 Limitations and future directions

The current diagnostic reliance on diaphragmatic excursion measurements highlights the need to incorporate more comprehensive ultrasonographic parameters, such as diaphragmatic thickness and thickening fraction, in future studies. Although traditional ISB remains safe for low-risk patients, the increasing age and comorbidity burden in contemporary surgical populations underscore the need for effective HDP-mitigation strategies. However, it must be acknowledged that ultrasound-guided techniques are inherently operator-dependent. Although all procedures in this study were performed by experienced anesthesiologists, a learning curve is associated with accurately identifying the fascial plane over the anterior scalene muscle and achieving consistent fluid distribution. This factor may affect the technique’s reproducibility among less experienced practitioners. Future efforts should therefore focus not only on refining the technique but also on developing standardized training protocols and competency assessments to facilitate its reliable adoption across diverse clinical settings. Future research should also aim to: (1) quantify the impact of HDP on rehabilitation adherence and hospital length of stay; (2) validate the efficacy of saline pre-injection in high-risk cohorts (e.g., patients with severe chronic obstructive pulmonary disease or morbid obesity); and (3) establish standardized ultrasonographic diagnostic criteria for HDP. Fourth, postoperative follow-up was limited to 48 h to assess early HDP and analgesia outcomes. Although this was suitable for evaluating the primary objective of HDP incidence, the short timeframe may have missed delayed respiratory or neurological sequelae. Future studies with extended follow-up periods are warranted to assess potential late effects of phrenic nerve involvement. Finally, as a single-center study with a limited sample size, our investigation did not permit stratified analysis based on patient baseline risk factors such as body mass index, pulmonary comorbidities, or age. Although baseline characteristics were well balanced between groups, we could not assess whether these variables modulate the impact of the intervention on diaphragmatic function and clinical outcomes. Consequently, future large-scale, multicenter trials are warranted to: (1) perform stratified analyses across distinct risk populations—including patients with severe chronic obstructive pulmonary disease or morbid obesity—to clarify the technique’s applicability in clinically relevant subgroups; and (2) validate its reproducibility and generalizability across diverse clinical environments.

## 5 Conclusion

The pre-injection of normal saline superficial to the ASM significantly reduced the incidence of HDP induced by ISB. This prophylactic saline injection technique may therefore expand ISB eligibility for patients with preexisting respiratory compromise, as it provides equivalent analgesic efficacy while preserving diaphragmatic function.

## Data Availability

The raw data supporting the conclusions of this article will be made available by the authors, without undue reservation.
